# E6-induced selective translation of WNT4 and JIP2 promotes the progression of cervical cancer via a noncanonical WNT signaling pathway

**DOI:** 10.1038/s41392-019-0060-y

**Published:** 2019-09-13

**Authors:** Lin Zhao, Longlong Wang, Chenglan Zhang, Ze Liu, Yongjun Piao, Jie Yan, Rong Xiang, Yuanqing Yao, Yi Shi

**Affiliations:** 1 School of Medicine, Nankai University, 94 Weijin Road, 300071 Tianjin, China; 2 Department of Obstetrics and Gynecology, Chinese PLA General Hospital, 28 Fuxing Road, 100853 Beijing, China

**Keywords:** Gynaecological cancer, Cancer genomics

## Abstract

mRNA translation reprogramming occurs frequently in many pathologies, including cancer and viral infection. It remains largely unknown whether viral-induced alterations in mRNA translation contribute to carcinogenesis. Most cervical cancer is caused by high-risk human papillomavirus infection, resulting in the malignant transformation of normal epithelial cells mainly via viral E6 and E7 oncoproteins. Here, we utilized polysome profiling and deep RNA sequencing to systematically evaluate E6-regulated mRNA translation in HPV18-infected cervical cancer cells. We found that silencing E6 can cause over a two-fold change in the translation efficiency of ~653 mRNAs, most likely in an eIF4E- and eIF2α-independent manner. In addition, we identified that E6 can selectively upregulate the translation of WNT4, JIP1, and JIP2, resulting in the activation of the noncanonical WNT/PCP/JNK pathway to promote cell proliferation in vitro and tumor growth in vivo. Ectopic expression of WNT4/JIP2 can effectively rescue the decreased cell proliferation caused by E6 silencing, strongly suggesting that the WNT4/JIP2 pathway mediates the role of E6 in promoting cell proliferation. Thus, our results revealed a novel oncogenic mechanism of E6 via regulating the translation of mRNAs.

## Introduction

Cervical carcinoma is the fourth leading cause of cancer death in women, affecting 570,000 women and resulting in 311,000 deaths in 2018 worldwide.^1^ Human papillomaviruses (HPVs), especially high-risk types such as type 16 and 18 (HPV16 and HPV18), are associated with most cervical carcinomas.^2^ The oncogenic properties of HPV16 and HPV18 depend mainly on viral E6 and E7 oncoproteins, which are able to induce many cellular alterations that are characteristics of cancer cells. It has been well established that E6 and E7 are able to respectively degrade and inactivate the tumor suppressors p53 and pRb, leading to deregulated cell cycle progression without the induction of cell apoptosis and senescence.^3,4^ In addition to p53-dependent mechanisms, E6 has been shown to stimulate more cancer-causing changes, including enhanced telomerase activity, tumor suppressor protein degradation, such as Scribble and Dlg, oncogenic miRNA regulation, and WNT signaling augmentation.^5–8^

Many efforts to explore the oncogenic mechanism of E6 have been focused on E6-regulated protein degradation and transcription. However, as an essential step in gene expression, mRNA translation control plays a crucial role in maintaining proteome homeostasis and controlling cellular phenotypes in response to many signals, such as nutrient supply, growth factors and stresses, including viral infection^9–12^; in fact, protein synthesis is a more energy-consuming process than mRNA transcription and accounts for ~50% of cellular energy. Therefore, controlling protein synthesis is an economical way for cells to adapt to stresses.^13,14^ HPV18 E6 was reported to induce the transcription of eukaryotic translation initiation factor 4E (eIF4E) and facilitate the dephosphorylation of the α subunit of eukaryotic translation initiation factor 2 (eIF2α), whose phosphorylation under various stresses leads to protein synthesis inhibition.^15,16^ Translation reprogramming caused by aberrant functions of translation factors, especially translation initiation factors, has been reported in several pathologies, including a variety of human malignancies. For instance, eIF4E has been found to be increased in many cancers and selectively increases the translation of a subset of mRNAs harboring long and structured 5′ UTRs or a 5′ terminal oligopyrimidine tract (5′TOP). Many of these mRNAs encode oncogenic proteins, including c-myc, cyclins, BCL-2, survivin, VEGF, and FGF.^17,18^

There have been no studies that systematically evaluate E6-induced reprogramming in mRNA translation and its significance in carcinogenesis. Here, we utilized polysome profiling followed by deep sequencing to globally analyze E6-regulated mRNA translation, i.e., the translatome. We found broader oncogenic pathways regulated by E6 through its regulation of the translatome. Among these pathways, we confirmed that E6 was able to activate the noncanonical WNT/PCP/JNK signaling pathway to promote the proliferation of cervical cancer cells in vitro and cervical cancer growth in vivo.

## Results

### E6 of HPV18 affects mRNA translation in HeLa cells

**Fig. 1:**
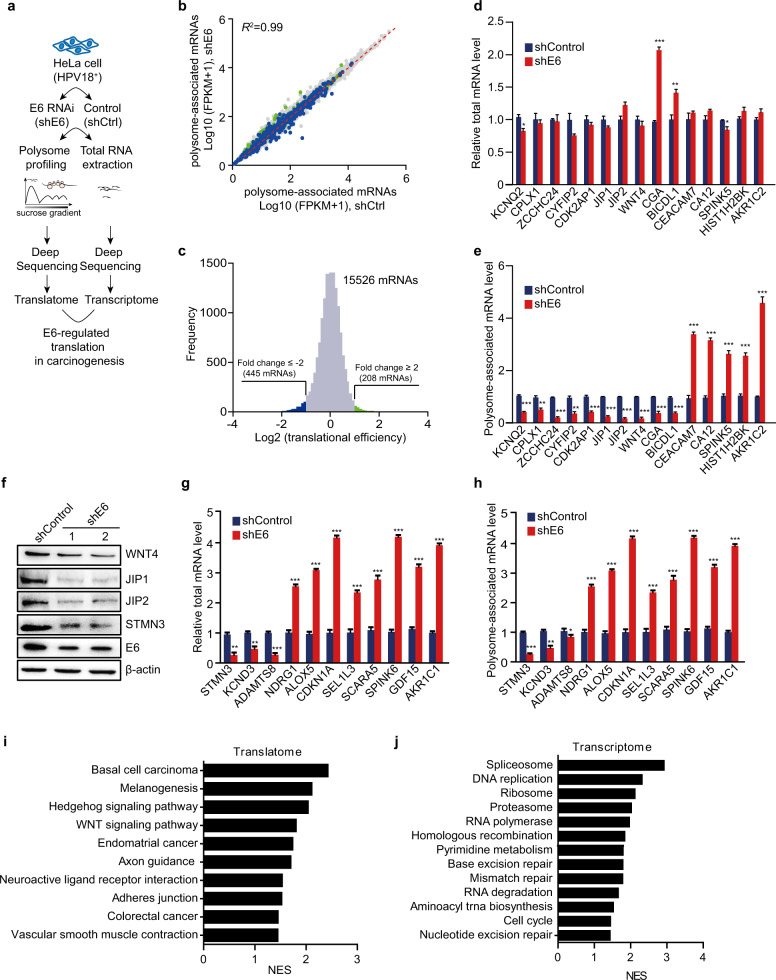
E6 of HPV18 affects mRNA translation in HeLa cells. **a** Schematic of the protocol for identifying E6-regulated mRNA translation at the transcriptome level. **b** Counts of polysome-associated mRNAs (fragments per kilobase per million mapped fragments, FPKMs) from E6-silenced (shE6) and control (shControl or shCtrl) cells. mRNAs with over a two-fold decrease or increase are indicated in blue and green, respectively. **c** Distribution of changes in translation efficiency for shControl and shE6 cells. mRNAs with decreased (fold change ≤ −2) or increased (fold change ≥ 2) translation efficiencies are indicated as above. **d**, **e** Some mRNAs with inconsistent changes between the transcription level and translation level were further confirmed by qRT-PCR. The transcription levels (**d**) and translation levels (**e**) were measured using total RNAs or polysome-associated mRNAs. The data represent means ± SEM from at least three independent experiments (*p < 0.05, **p < 0.01, ***p < 0.001). **f** The protein levels of the indicated genes in shE6 and shControl HeLa cells were determined by western blot. **g**, **h** The transcription levels (**g**) and translation levels (**h**) of the indicated mRNAs with consistent changes were measured by qRT-PCR as described in **d** and **e**. The data represent means ± SEM from at least three independent experiments (*p < 0.05, **p *<* 0.01, ***p < 0.001). **i**, **j** Top pathways (E6-activated pathways) enriched in shControl HeLa cells from GSEA of RNA-Seq data for polysome-associated mRNAs (**i**) or the transcriptome (**j**)

To systematically evaluate whether HPV18 E6 caused alterations in the translation efficiency of mRNAs, we utilized polysome profiling followed by deep RNA sequencing, which provides a transcriptome-scale measurement of mRNA translation, in HeLa cells stably transfected with an E6-silencing shRNA (shE6) or shRNA targeting the unrelated LacZ gene as a control (shControl) (Fig. 1a and Supplementary Fig. S1a, b). Using sucrose-gradient centrifugation, efficiently translated mRNAs associated with polysomes were separated from untranslated free mRNAs (Supplementary Fig. S1c, d), which was further confirmed by the enrichment of mRNAs encoding β-actin, an actively expressed housekeeping protein, in the polysome fractions (Supplementary Fig. S1e).

The polysome-associated mRNAs from shE6 and shControl cells were further analyzed by deep sequencing. We detected 24 million exon-mapped ribosome-bound reads that corresponded to 15,526 actively translated mRNAs (Supplementary Table S2 and Fig. 1b). The proportion of one transcript can be reflected by its specific fragments per kilobase per million mapped fragments. To identify the mRNAs regulated by E6 at the translation level, we calculated shE6-induced changes in the translation efficiency of each mRNA after normalization to the total amount of that mRNA quantified by deep sequencing of the total RNA (Fig. 1a–c and Supplementary Table S2). We identified 445 decreased and 208 increased mRNAs with over a two-fold change (Fig. 1c).

To further confirm these results, we quantified the transcription and translation levels of some genes by quantitative RT-PCR and western blot (Fig. 1d–h). Based on the deep sequencing results, we first selected some of the genes showing great changes in their translation levels, but with no or little changes in their transcription levels. Quantitative RT-PCR using total RNA or polysome-associated RNA clearly showed the inconsistency between the transcription levels and the translation levels of these genes (Fig. 1d, e). We also tested the amount of some proteins encoded by the genes with little transcriptional changes, such as WNT4, JIP1 (or MAPK8IP1), and JIP2 (or MAPK8IP2) (Fig. 1d). Consistently, we observed a dramatic decrease in their protein levels when E6 was silenced (Fig. 1f). Some genes with increased transcription, such as CGA and BICDL1, even showed decreased translation (Fig. 1d, e). We also selected some genes showing consistent changes between their transcription and translation levels and confirmed the results using qRT-PCR (Fig. 1g, h). Among them, we chose STMN3 as an example to be further confirmed by western blot (Fig. 1h). Taken together, these results suggested that E6 was able to selectively regulate the translation of some genes.

E6 was shown to activate mTORC1 to stimulate 4E-BP1 hyperphosphorylation and eIF4E release, resulting in the assembly of the eIF4F complex and the initiation of protein synthesis in a 5′-cap-dependent manner.^19^ However, we found that knocking down E6 did not affect the translation efficiencies of the mRNAs with long and complex 5′ untranslated regions whose translation is eIF4E-dependent, such as MYC (log_2_(FC) = 0.19), VEGFA (log_2_(FC) = −0.30), Cyclin D1 (log_2_(FC) = 0.52), and Cyclin D3 (log_2_(FC) = −0.07) (Supplementary Table S2). In our dataset, the mRNAs harboring a 5′ terminal oligopyrimidine tract (5′ TOP) or 5′TOP-like motif, which are thought to be regulated by the mTORC1/eIF4E pathway as well,^20,21^ were not significantly affected by shE6 (Supplementary Table S3).

Next, we compared the transcriptome-based and translatome-based gene enrichment results to evaluate the global biological functions of E6. KEGG functional enrichment analysis and gene set enrichment analysis (GSEA) between the shE6 and shControl groups revealed that the translatome-based analysis showed broader oncogenic mechanisms of E6 (Fig. 1i), whereas the transcriptiome-based analysis suggested only confined effects of E6 on nucleic acid metabolism and DNA repair (Fig. 1j). Due to its better correlation with real protein levels, translatome-based analysis of gene function might be a more accurate method.

### E6 of HPV18 enhances the noncanonical WNT/PCP/JNK pathway by increasing the translation of WNT4 and JIP2

**Fig. 2:**
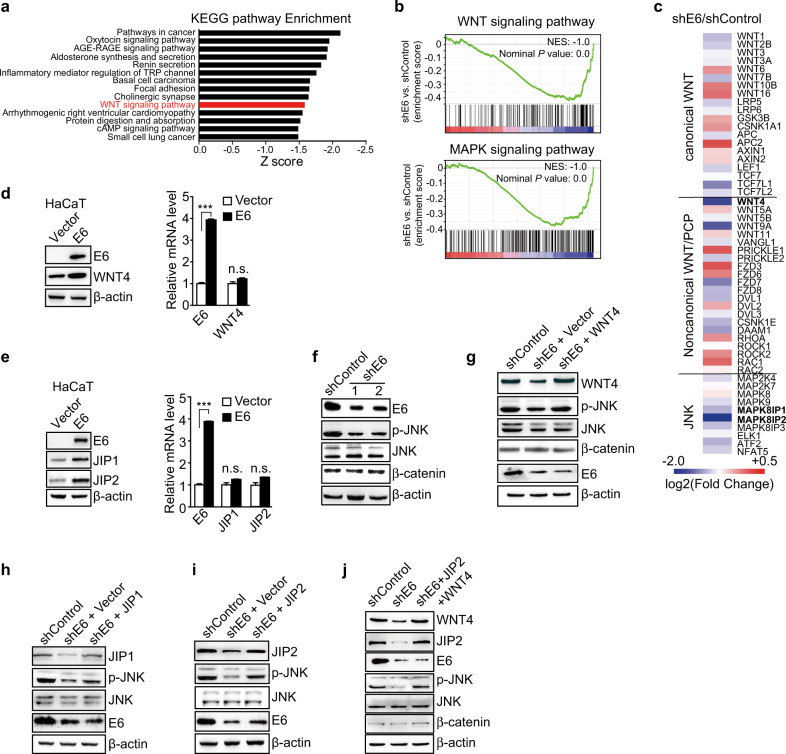
E6 of HPV18 activates the noncanonical WNT/PCP/JNK pathway by increasing the translation of WNT4 and JIP2. **a** Top KEGG pathways (E6-activated pathways) enriched in shControl HeLa cells based on the translation efficiency. **b** GSEA plot of the WNT and MAPK pathways based on the shE6 versus shControl translation efficiency profiles. NES, normalized enrichment score; *p*, nominal value. **c** Heatmap of the enriched genes found by genome-wide translation profiling; the results illustrate the changes in translation efficiencies of the indicated pathways. The translation levels shown are representatives of the mean log2 values from three replicates. The red signal denotes increased translation, and the blue signal denotes decreased translation in shE6 cells. **d**, **e** Ectopic expression of E6 in HaCaT cells increased the protein levels of WNT4 (**d**, left panel), JIP1 and JIP2 (**e**, left panel) without affecting their mRNA levels as examined by qRT-PCR (right panels in **d** and **e**). The data represent means ± SEM from at least three independent experiments (***p < 0.001, n.s. denotes no statistical significance). **f** E6-silenced (shE6, with two shRNAs targeting two different sites) and control (shControl) HeLa cells were analyzed by western blot for the indicated proteins. **g** WNT4 or empty vector control (vector) was ectopically expressed in shE6 HeLa cells, and the cells were analyzed by western blot for the indicated proteins. **h** Western blot showing that the ectopic expression of JIP1 in shE6 HeLa cells did not rescue the phosphorylation of JNK. **i** Western blot showing that the ectopic expression of JIP2 in shE6 HeLa cells could partially rescue JNK phosphorylation. **j** Western blot showing that the ectopic expression of both WNT4 and JIP2 greatly restored JNK phosphorylation

To evaluate the biological significance of E6-regulated translation, we utilized KEGG and GSEA functional enrichment analyses based on changes in translation efficiency (Supplementary Table S2). Many genes with more than a two-fold change in translation efficiency were enriched in cancer-related pathways (Fig. 2a). GSEA analysis revealed a significant downregulation of genes that are involved in the noncanonical WNT/PCP and MAPK/JNK pathways (Fig. 2b, c).

Next, we selected the most affected target genes involved in the WNT/JNK pathways, i.e., WNT4, JIP1, and JIP2 (Fig. 1d–f), to further confirm E6-regulated translation of these genes. We observed a shift in the transcripts of WNT4, JIP1, and JIP2 from the polysome fraction to the free mRNA fraction in E6-silenced HeLa cells (Supplementary Fig. S2a–c), while there was no such shift in the transcript levels of STMN3, whose translation was not affected by E6 (Fig. 1f–h and Supplementary Fig. S2d). To further confirm these results, we overexpressed E6 in HaCaT cells, which are keratinocyte cells without HPV infection.^22^ Consistently, E6 was able to enhance the translation of WNT4, JIP1, and JIP2 without affecting their transcription in HaCaT cells (Fig. 2d, e).

To clarify which WNT signaling pathway was affected by E6 through translation regulation, we firstly examined the functions of the major E6 targets WNT4, JIP1, and JIP2 in HeLa cells. Knocking down WNT4 significantly decreased the phosphorylation of JNK without affecting β-catenin, suggesting that WNT4 activates the β-catenin-independent noncanonical WNT–PCP–JNK pathway in this context (Supplementary Fig. S3a, b). As the key downstream scaffold protein that facilitates the MAPK/JNK pathway, JIP2, but not JIP1,^23–25^ was responsible for the activation of JNK in HeLa cells (Supplementary Fig. S3c–f). Consistently, knocking down E6 (shE6) significantly inhibited JNK phosphorylation without affecting the level of β-catenin (Fig. 2f). Separate or simultaneous restoration of WNT4 and JIP2, but not JIP1, in shE6 cells can restore the phosphorylation of JNK (Fig. 2g–j), strongly suggesting that E6 activates the WNT/PCP/JNK pathway by enhancing the translation of WNT4 and JIP2.

### E6 promotes cell proliferation through translational activation of the WNT/PCP/JNK pathway

**Fig. 3:**
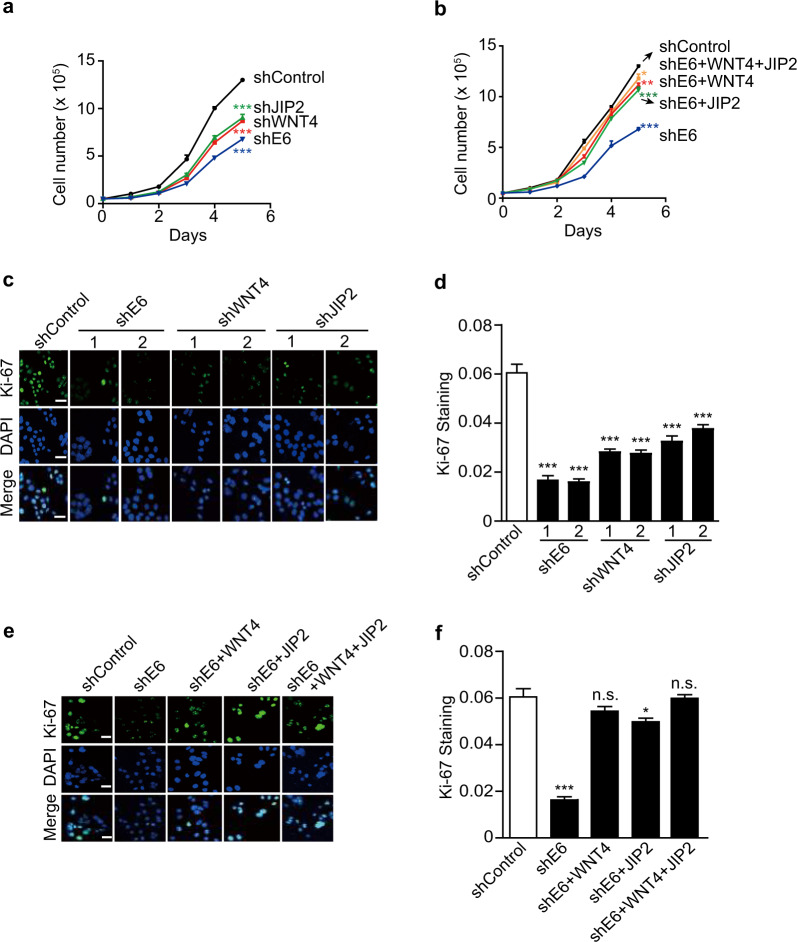
E6 promotes cell proliferation through translational activation of the WNT/PCP/JNK pathway. **a**, **b** Growth curves of HeLa cells infected with lentivirus expressing the indicated shRNAs (indicated by ‘sh’) or genes (indicated by gene symbols). The data represent means ± SEM from at least three independent experiments (*p < 0.05, **p *<* 0.01, ***p < 0.001). **c–f** Actively proliferating HeLa cells infected with lentivirus expressing the indicated shRNAs or genes are shown by immunofluorescent staining for Ki-67. Representative images are shown in **c** and **e**, and the quantification of Ki-67 signals is plotted in **d** and **f**. ‘n.s.’ denotes ‘not significant’. The scale bars represent 25 μm. The data represent means ± SEM from at least three independent experiments (*p < 0.05, ***p < 0.001)

JNK kinases are well-established regulators of cell cycle progression, and their activation affects cell proliferation in a cell context-dependent manner.^26^ Next, we explored the effect of E6-induced activation of the WNT/PCP/JNK pathway on the proliferation of cervical cancer cells. Knocking down E6 significantly inhibited the proliferation of HeLa cells, as expected (Fig. 3a). Silencing WNT4 or JIP2 alone was able to inhibit the proliferation of HeLa cells as well, but to a lesser extent, suggesting a pro-proliferative role of the WNT4/JIP2/JNK pathway in HeLa cells (Fig. 3a). Ectopic expression of WNT4 and JIP2 separately or simultaneously in E6-silenced HeLa cells could partially or largely restore cell proliferation (Fig. 3b). To further confirm this finding, we determined the Ki-67 level, which reflects active cell proliferation.^27^ Consistently, we observed fewer Ki-67-positive cells when E6, WNT4, and JIP2 were knocked down (Fig. 3c, d and Supplementary Fig. S4a–c). The ectopic expression of WNT4 and JIP2 in E6-silenced HeLa cells rescued the number of Ki-67-positive cells (Fig. 3e, f). The better rescue effects observed upon ectopic expression of both WNT4 and JIP2 strongly suggested that the WNT4/JIP2 pathway mediated the effect of E6 on promoting cervical cancer cell proliferation (Fig. 3e, f).

### E6 promotes tumor growth via the WNT/PCP/JNK pathway in vivo

**Fig. 4:**
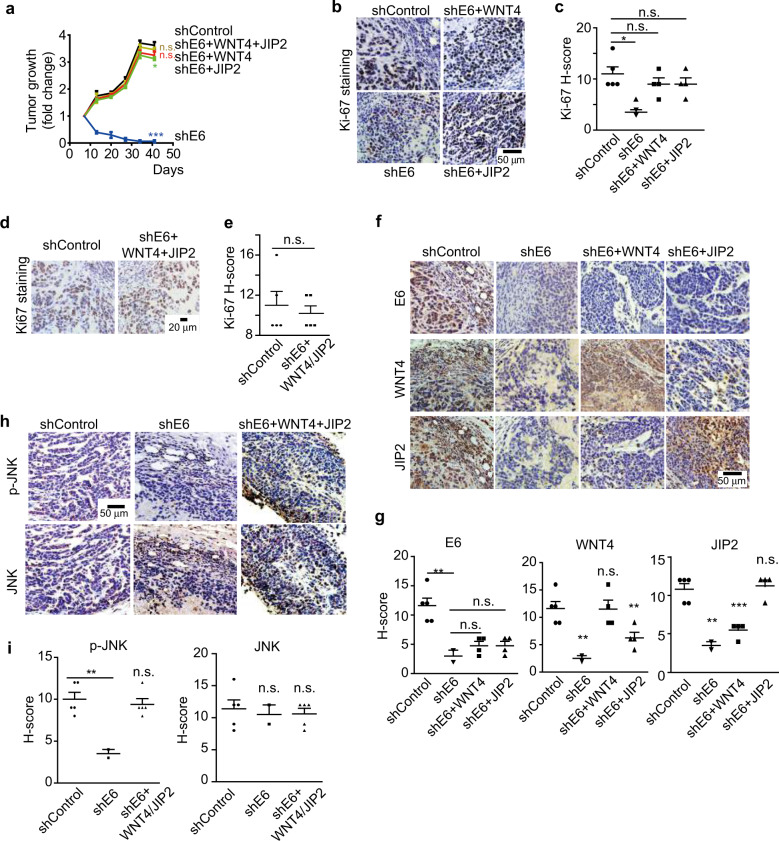
E6 promotes tumor growth through the activation of WNT/PCP/JNK pathway. **a** HeLa cells were stably transfected with the indicated shRNA or genes and injected subcutaneously into NOD/SCID mice. The growth of the tumor xenografts is plotted (*n* = 4–5). ‘n.s.’ denotes ‘not significant’, *p < 0.05. **b–e** Immunohistochemistry (IHC) staining for Ki-67 to show actively proliferating tumor cells in the xenografts. Representative images are shown in **b** and **d**. Quantitative results based on the *H*-score are plotted in **c** and **e**. ‘n.s.’ denotes ‘not significant’, *p < 0.05. **f**, **g** IHC staining of E6, WNT4, and JIP2 proteins in the indicated xenografts (**f**), and the protein levels were quantified based on the *H*-score (**g**). ‘n.s.’ denotes ‘not significant’, **p < 0.01, ***p < 0.001. **h**, **i** IHC staining for phosphorylated JNK (p-JNK) to show JNK activation in the indicated xenografts (**h**). The *H*-score-based quantification results are shown in **i**. ‘n.s.’ denotes ‘not significant’, **p < 0.01

To gain insights into the significance of the E6-regulated WNT/PCP/JNK pathway in vivo, we performed a xenograft experiment using HeLa cells. Tumor growth inhibition induced by silencing E6 could be largely restored by ectopic expression of either WNT4 or JIP2, which was shown by measuring the tumor growth curve (Fig. 4a and Supplementary Fig. S4d) and Ki-67-positive cells (Fig. 4b, c). The best rescue of tumor growth was observed when both WNT4 and JIP2 were ectopically expressed (Fig. 4a, d, e and Supplementary Fig. S4e). In tumor xenografts, silencing E6 resulted in reduced protein amounts of WNT4 and JIP2, as shown by immunohistochemistry (IHC), which was consistent with what we observed in HeLa cells cultured in vitro (Fig. 4f, g). We also observed that silencing E6-induced JNK inactivation, which could be rescued by ectopic expression of WNT4 and JIP2 (Fig. 4h, i).

### E6/WNT/JNK pathway in human cervical adenocarcinoma (CAC)

**Fig. 5:**
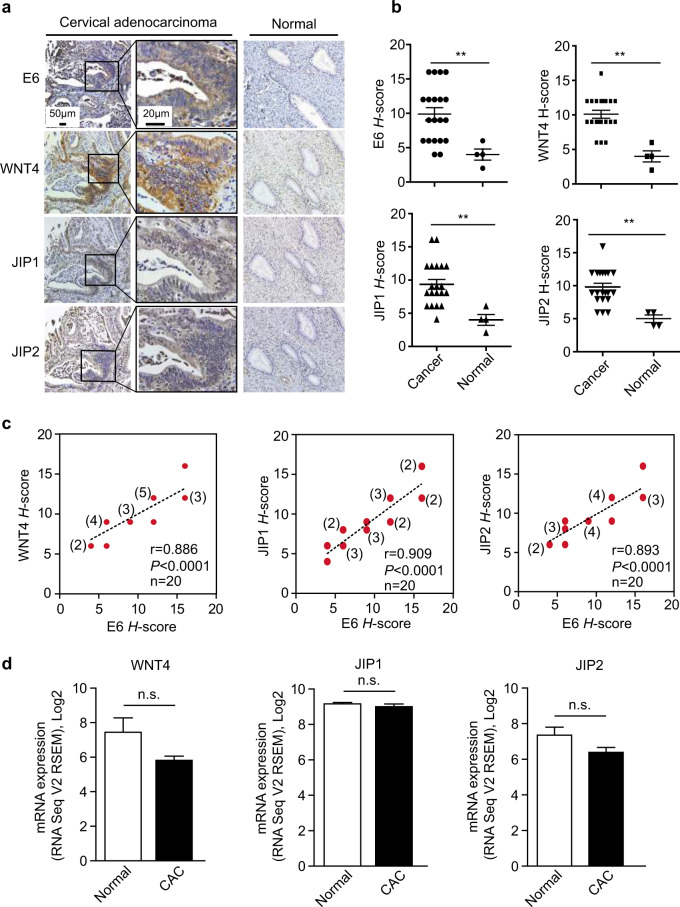
E6 correlates with the WNT4/JIP2/JNK pathway in human cervical adenocarcinoma (CAC). **a**, **b** IHC staining of the indicated proteins in a human CAC tissue array containing 20 intact cancer tissues and 4 normal adjacent tissues (NATs) or normal cervical tissues. Representative images are shown in **a**, and the *H*-score-based quantification results are shown in **b**. **p < 0.01. **c** Staining scores from **b** were subjected to correlation analyses for E6 and WNT4, JIP1 or JIP2. **d** The mRNA expression levels of WNT4, JIP1, and JIP2 in the cancer cells of 52 CAC patients and 3 NAT/normal cervical tissues from TCGA are plotted as bar graphs. The data represent means ± SEM. ‘n.s.’ denotes ‘not significant’

To gain further insights into the significance of the E6-regulated WNT4/JIP2/JNK pathway in patients, we assessed the protein levels of E6, WNT4, and JIP2 by IHC using tissue arrays containing 20 human CAC samples and 4 normal cervical samples (two normal tissues and two cancer-adjacent normal tissues). We found very low levels of staining for WNT4, JIP2, and JIP1 in E6-negative normal cervical tissue, whereas high levels of WNT4, JIP2, and JIP1 were detected in E6-positive CAC samples (Fig. 5a). We quantified IHC staining in CAC specimens with a scoring scale (*H*-score), which combined the staining intensity and the percentage of positive cells. There were significant differences between the *H*-scores of normal and CAC samples for the levels of WNT4, JIP2, and JIP1 (Fig. 5b). Notably, the E6 protein level showed a good positive correlation with the levels of WNT4, JIP2, and JIP1 (Fig. 5c), strongly supporting E6 regulation on the WNT4/JIP2/JNK pathway in CAC patients.

We also analyzed the transcriptome of 52 CAC patient tissue samples and 2 cancer-adjacent normal tissue samples obtained from The Cancer Genome Atlas. The mRNA levels of WNT4, JIP1, and JIP2 did not show significant differences between the normal samples and CAC samples (Fig. 5d), suggesting the limitation of transcriptome analysis in finding new molecular mechanisms underlying cervical carcinogenesis.

## Discussion

Gene expression is regulated not only at the transcription and post-transcription levels (for example, microRNA and siRNA) but also at the translation level, which is important for protein-encoding genes and has recently attracted growing interest.^28–31^ Translation control has been found to occur in response to many stresses and pathologies, including viral infection and cancer.^32,33^ However, until now, most mechanistic studies on the roles of the main oncogenic protein of high-risk HPV, E6, were focused on E6-induced transcription changes and protein degradation.^34,35^ Although some studies have revealed that E6 can affect the translation initiation factors eIF4E and eIF2α^15,16^ and may thus have potential roles in regulating translation, there have been no studies globally exploring the E6-regulated translatome and its roles in carcinogenesis. To more accurately and broadly examine protein production affected by E6, we utilized polysome profiling followed by deep sequencing in this study. Our results clearly showed that E6 could affect the translation of 653 mRNAs (with a greater than two-fold change). We also revealed that E6, through translation regulation, activates the noncanonical WNT/PCP/JNK pathway to promote the proliferation of cervical cancer cells and tumor growth.

Current studies on translation regulation are focused on the rate-limiting initiation step. Deregulated translation initiation factors that cause translation reprogramming promote a variety of cancer phenotypes, including proliferation, survival, angiogenesis and metastasis.^36,37^ E6 was reported to activate the mTORC1/eIF4E pathway, which is especially important for the translation of many oncoprotein-encoding mRNAs harboring long and structured 5′UTRs (e.g., c-Myc and VEGF) and mRNAs with 5′TOP or 5′TOP-like motifs.^19,21^ However, silencing E6 did not result in significant changes in the translation efficiencies of these sets of mRNAs (Supplementary Table S3), suggesting that E6 might regulate mRNA translation in an eIF4E-independent manner. E6 has also been reported to promote the dephosphorylation of eIF2α. When phosphorylated in response to stresses, e.g., lack of nutrients and oxygen, eIF2α stimulates the translation of mRNAs with short upstream ORFs (uORFs) that encode proteins involved in stress responses, such as ATF4 and DDIT3.^38–40^ After silencing E6, we did not observe changes in the translation efficiencies of these mRNAs (Supplementary Table S2), suggesting that E6-mediated translation does not occur through eIF2α. Taken together, our results indicated that E6 might regulate mRNA translation through an unknown mechanism, and our data here provide a useful tool for the discovery of a novel mechanism underlying translation regulation.

Increasing evidence shows that WNT signaling contributes to the initiation, progression, invasion, and drug resistance of cervical cancers.^41–43^ However, it remains unknown exactly how the WNT signaling pathway is involved. Hava Lichtig et al. reported that E6 of HPV16 was able to augment the WNT/β-catenin/TCF-dependent signaling response without altering the level and distribution of β-catenin. They found that E6-induced activation of TCF/LEF, downstream transcription factors of the WNT/β-catenin/TCF signaling pathway, required the association of the ubiquitin ligase E6AP with E6.^6^ Consistently, we also found that silencing E6 did not change the level of β-catenin (Fig. 2f). In addition, our results showed that silencing E6 reduced the translation of TCF7L1, TCF7L2, and LEF1 (Fig. 2c and Supplementary Table S2), suggesting that E6 might directly increase the translation efficiency of the TCF/LEF family of transcription factors to mimic the activation of canonical WNT signaling. More importantly, we revealed that through regulating mRNA translation, E6 could dramatically activate noncanonical, β-catenin-independent WNT/PCP/JNK signaling (Fig. 2f), which is much less studied in carcinogenesis. By increasing the translation of WNT4 and JIP2, E6 can activate the WNT4-induced noncanonical pathway, and JIP2 facilitates the activation of JNK downstream to promote the proliferation of cervical cancer cells and tumor growth. Taken together, our data revealed that high-risk HPV infection-induced translational reprogramming can promote carcinogenesis. In our results, WNT4 and JIP2 dramatically rescued the role of E6 in promoting cancer cell proliferation both in vitro (Fig. 3b, e, f) and in vivo (Fig. 4a–e), suggesting that the β-catenin-independent, noncanonical WNT/PCP/JNK pathway, especially WNT4 and JIP2, could be an ideal target for treating HPV-induced cervical cancer.

We also found that JIP1 was translationally regulated by E6 (Figs. 1f, 2c, e). However, JIP1 showed marginal effects on the E6-induced phosphorylation of JNK when compared with that of JIP2 (Fig. 2h, i and Supplementary Fig. S3d, f), suggesting that JIP1 was not a key downstream player of E6 in promoting the progression of cervical cancer. It has been reported that excess JIP1 may decrease JNK activity through a negative feedback loop by sequestering JNK in the cytoplasm, preventing the activation of c-Jun and ATF2.^44^ Thus, there might be another mechanism to suppress JIP1.

## Materials and methods

### Cell culture

HeLa cells (derived from cervical cancer) were cultured in DMEM supplemented with 10% (v/v) fetal bovine serum and antibiotics (penicillin and streptomycin). Cells were grown in an atmosphere of 5% CO_2_ at 37 °C.

### Vector construction and virus production

shRNA templates targeting E6, WNT4, JIP1, and JIP2 were cloned into the lentivirus-based plasmid pLV-H1-EF1α-puro (Biosettia Inc., USA). Lentivirus infections were performed following the manufacturer’s instructions. Stable cell lines were selected by adding puromycin (2.5 μg/mL) to the cell culture medium.

The coding sequences for HPV18 E6, WNT4, JIP1, and JIP2 were amplified from HeLa mRNAs by reverse-transcription PCR (RT-PCR) using a TransStart FastPfu DNA Polymerase Kit (TransGen Biotech, China) and inserted into the lentivirus-based plasmid pLV-EF1α-MCS-IRES-Bsd (Biosettia Inc., USA). All plasmids were verified by sequencing. The primer sequences are listed in Supplementary Table S1. Lentiviruses were produced in HEK293T cells as described previously.^45^

### RNA extraction and RT-qPCR

Cells were washed three times with ice-cold PBS. Total RNA was extracted using TRIzol reagent (Invitrogen Inc., USA) and reverse transcribed into complementary DNA with the First-Strand cDNA Synthesis System (TransGen Biotech, China). SYBR Green-based real-time quantitative PCR (qPCR) analysis was performed using a LightCycler^®^96 system (Roche). The primer sequences are listed in Supplementary Table S1. The 2^−ΔΔCt^ method was utilized to calculate the relative fold change of each gene with β-actin as the normalization control.

### Polysome profiling

Polysome profiling was performed as described previously by Seimetz et al.^46,47^ First, sucrose gradient preparation was performed as follows: 15, 17.5, 20, 22.5, 27, 30, 32.5, 35, 37.5%, and 40% sucrose stock solutions in a buffer containing 20 mM Tris (pH 7.4), 150 mM KCl, 5 mM MgCl, 1 mM DTT, and 100 μg/ml cycloheximide (CHX) were created. A discontinuous sucrose density gradient was prepared by layering successive decreasing sucrose density solutions upon one another. The gradients were handled with care to avoid disturbance and air bubble introduction. For cell lysis, HeLa cells (~70–80% confluent in a 10-cm dish) were treated with 100 µg/ml CHX for 20 min before harvesting. After being washed twice with PBS containing 100 µg/ml CHX, the cells were scraped into 10 ml of ice-cold PBS containing 100 µg/ml CHX and centrifuged at 600 × *g* for 5 min at 4 °C to collect the cell pellet. Then, the cells were lysed in 200 µl of polysome lysis buffer (20 mM Tris pH 7.4, 150 mM KCl, 5 mM MgCl, 1 mM DTT, 100 μg/ml CHX, 0.5% NP-40, and 40 U/ml RNase inhibitor) for 20 min on ice. Next, the cells were centrifuged at 10,000 × *g* for 20 min at 4 °C to collect the supernatants. Third, ultracentrifugation and fraction collection were performed. The cell lysates were carefully loaded on top of the sucrose gradient in ultracentrifugation tubes without disturbing the gradient. Ultracentrifugation was performed in an SW-41Ti rotor at 111,000 × *g* for 4 h at 4 °C. The sucrose gradient was separated into fourteen 0.75-ml fractions and gently transferred to 1.5 ml tubes. The OD at 254 nm was measured for each fraction to determine which fractions contain the polysome. Last, RNA isolation, deep RNA sequencing, and RT-qPCR were performed. A total of 750 µl of phenol–chloroform (1:1) was added to each fraction and vortexed. After centrifugation at 13,000 rpm for 15 min at 4 °C, the supernatants were transferred to new tubes, and an equal volume of isopropanol was added to precipitate the RNA. RNA pellets were washed once with ice-cold 75% ethanol before being dissolved in 20 µl of nuclease-free water.

The quality of the RNA was determined using an Agilent 2100 Bioanalyzer, and samples with RNA integrity numbers (RINs) over eight were used to construct the libraries and sequenced on BGISEQ-500 platforms.^48,49^

One microgram of RNA was used to synthesize cDNA using Abm’s 5x All-In-One RT MasterMix (Abm, Canada). Each quantitative PCR (qPCR) reaction was set up with 2 μL of cDNA products and SYBR Green PCR mix (TransGen Biotech, China). The primer sequences are listed in Supplementary Table S1.

### Protein extraction and western blot

Total cellular proteins were prepared in RIPA lysis buffer with phosphatase inhibitor cocktail and protease inhibitor cocktail (Sigma-Aldrich, St Louis, MO, USA). Twenty micrograms of total proteins were loaded into 12% Tris-acrylamide gels. The antibodies used in our studies were anti-β-actin (Santa Cruz, sc-47778), anti-phospho-JNK (Thr183/Tyr185) (Wanlei, H01291813), anti-JNK (Santa Cruz, sc-571), anti-β-catenin (Cell Signaling Technology, #8480), anti-P53 (Santa Cruz, sc-126), anti-HPV18 E6 (Santa Cruz, sc-365089), anti-WNT4 (Santa Cruz, sc-376279), anti-JIP1 (Abcam, ab24449), anti-JIP2 (Santa Cruz, sc-53553), and anti-STMN3 (Abcam, ab171625).

### Immunofluorescent staining and confocal microscopy

Cells were fixed in 4% paraformaldehyde followed by blocking with 5% goat serum in PBS. The cells were then incubated with an anti-Ki-67 antibody at a dilution of 1:200 (Abcam, ab16667, Cambridge, UK). After washing, the cells were incubated with secondary antibodies conjugated with Alexa Fluor-488 (Fisher-Thermo, USA) and counterstained with DAPI. Images of the cells were taken using an Olympus FV1000 confocal microscope (40 × oil objective) (Olympus, Japan).

### Immunohistochemistry

Consecutive sections of a human cervical cancer tissue array containing 20 intact cervical adenocarcinoma tissues, 2 normal cervical tissue and 2 normal adjacent cervical tissues were purchased from Alenabio (CR246). The sections were stained with anti-WNT4 antibody (Santa Cruz, sc-376279) at a 1:200 dilution, anti-JIP1/JIP2 antibody (Absin, #113309 and #133562, respectively) at a 1:100 dilution and anti-E6 antibody (Santa Cruz, sc-57835) at a 1:400 dilution. After washing, the sections were incubated with biotin-conjugated secondary antibodies, followed by streptavidin-HRP; the sections were finally visualized with 3,3′-diaminobenzidine (DAB) substrate. Images were taken with an Olympus BX53 microscope under a 20× objective (Olympus Co, Tokyo, Japan). Immunostaining was also performed on tumor xenograft sections using a similar procedure. The *H*-score was used for quantifying E6, WNT4, JIP1, and JIP2 in normal and tumor tissues, and this score was calculated by multiplying the staining area (scored as 1, 2, 3, and 4; 1 for 0–25%, 2 for 25–50%, 3 for 50–75%, and 4 for 75–100% positively stained area) with the staining intensity (negative, weak, moderate, and strong were scored as 1, 2, 3, and 4 based on color density). Student’s *t*-test was performed for statistical analysis.

### Cell proliferation assay

A total of 5 × 10^4^ cells were seeded in each well of a six-well plate on day 0, and the numbers of live cells were counted daily using a hemocytometer after trypan blue staining. The data are from three biologically independent experiments.

### Mouse xenograft cervical cancer model

HeLa cells (5 × 10^6^) were injected subcutaneously into six-week-old female NOD/SCID mice. Tumor sizes were measured by a caliper, and tumor volumes were calculated using the formula (length × width^2^)/2.

### Statistical analysis

Statistical analysis was performed using GraphPad software. Student’s *t*-test was used to evaluate the differences between experimental groups. A *P* value < 0.05 was considered statistically significant. Statistical significance is indicated as follows: *p < 0.05, **p < 0.01, and ***p < 0.001. All experiments were performed at least three times.

## Supplementary information

Supplementary Information

Supplementary Table
