# Supplementary material for: E6-induced selective translation of WNT4 and JIP2 promotes the progression of cervical cancer via a noncanonical WNT signaling pathway
Source: Signal Transduct Target Ther. 2019 Sep 13;4:32. doi: 10.1038/s41392-019-0060-y (PMC6799841; doi:10.1038/s41392-019-0060-y)
Supplement: Supplementary file 1 — Supplementary Information [file 41392_2019_60_MOESM1_ESM.docx]

**SupplementaryInformation**

(This file contains supplementary figures S1-S4, supplementary table S1 and table S3, table S2 is provided separately.)

**E6-induced selective translation of WNT4 and JIP2 promotes the progression of cervical cancer via a non-canonical WNT signaling pathway**

Lin Zhao^1, 2^, Longlong Wang^1^, Chenglan Zhang^1^, Ze Liu^1^, Yongjun Piao^1^, Jie Yan^1^, Rong Xiang^1*^, Yuanqing Yao^1, 2*^ and Yi Shi^1*^

^1^School of Medicine, Nankai University, 94 Weijin Road, Tianjin 300071, China

^2^Department of Obstetrics and Gynecology, Chinese PLA General Hospital, 28 Fuxing Road, Beijing 100853, China

*Correspondence to: Yi Shi ([yishi@nankai.edu.cn](mailto:yishi@nankai.edu.cn)), Yuanqing Yao ([yqyao@126.com](mailto:yqyao@126.com)) and Rong Xiang ([rxiang@nankai.edu.cn](mailto:rxiang@nankai.edu.cn))
Phone: (86)-22-23509482

Fax:(86)-22-23502554

**Figure S1**


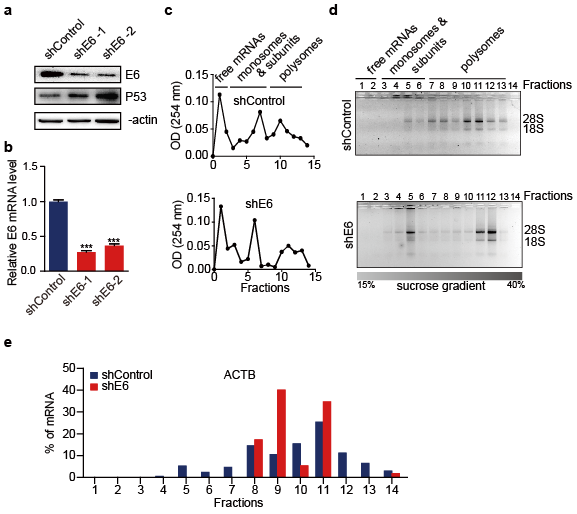


**FigureS1. Isolation of polysome-associated mRNAs**

**(a) and (b)** E6-silencing (shE6) efficiency in HeLa cells was examined by Western blot (a) and qRT-PCR (b). As reported, silencing E6 caused stabilization of p53 was observed, further confirming the efficiency of two E6 shRNAs. Data represents means ± SEM from at least three independent experiments (****p*< 0.001).

**(c)** After sucrose gradient centrifugation, the absorbance at 254 nM of each fraction was measured to define polysome-containing fractions.

**(d)** RNA isolated from each fraction was analyzed by 1% agarose gel electrophoresis followed by EB staining to determine the polysome-containing fractions, indicated by 28S and 18S rRNAs.

**(e)** β-actin (ACTB) mRNA abundance in fractions from **(d)** was determined by qRT-PCR and calculated as a percentage of the total in all fractions.

**Figure S2**


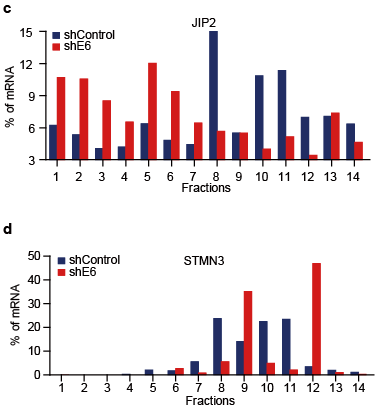

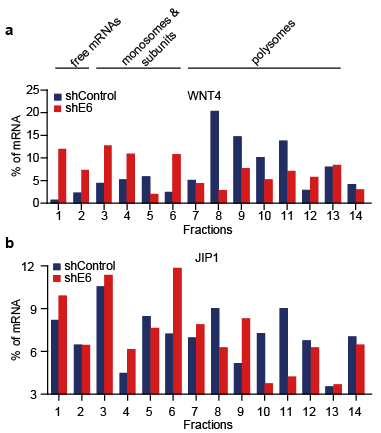


**FigureS2. Silencing E6 selectively affected the translation of some mRNAs**

**(a-d)** mRNA abundances of WNT4 (a), JIP1 (b), JIP2 (c) and STMN3 (d) in fractions (as described in Supplementary Fig. S1d and S1e) were quantified by qRT-PCR, and calculated as percentages of the total in all fractions.

**Figure S3**


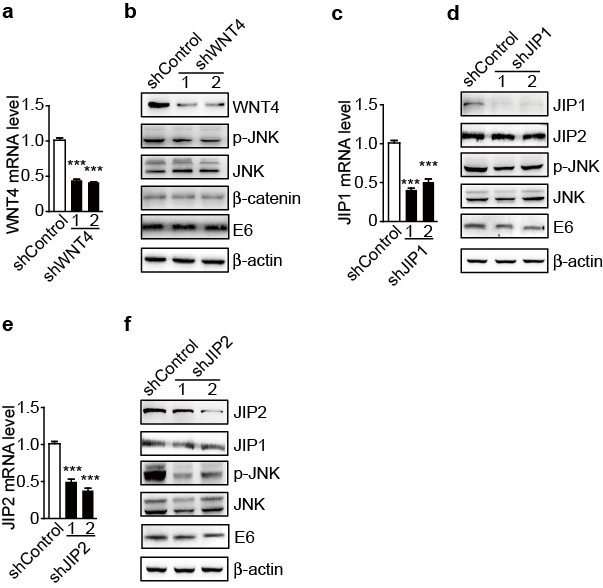


**FigureS3. Activated WNT4/JIP2/JNK pathway in HeLa cells**

**(a)** The silencing efficiencies of two shRNAs targeting WNT4 (shWNT4) in HeLa cells were examined by qRT-PCR.

**(b)** Silencing WNT4 caused effects on canonical WNT/β-catenin and non-canonical WNT/JNK pathways were analyzed by western blot.

**(c) and (d)** The silencing efficiencies of two shRNAs targeting JIP1 (shJIP1) and the effects on JNK activation were analyzed by qRT-PCR (c) and western blot (d).

**(e) and (f)** The silencing efficiencies of two shRNAs targeting JIP2 (shJIP2) and the effects on JNK activation were analyzed by qRT-PCR (e) and western blot (f).

Data represents means ± SEM from at least three independent experiments (****p*< 0.001).

**Figure S4**


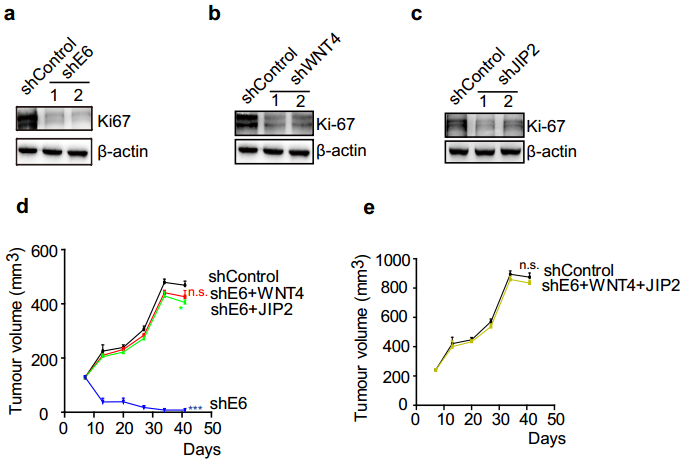


**FigureS4. Silencing WNT4/JIP2 inhibits the proliferation of HeLa cells**

**(a-c)** Silencing E6 (a), WNT4 (b) or JIP2 (c) caused reduction of Ki-67 protein levels were determined by western blot.

**(d) and (e)** HeLa cells were stably transfected with indicated shRNA or genes and subcutaneously injected into NOD/SCID mice. The volume of the tumor xenografts was plotted (n=4-5). ‘n.s.’ denotes ‘not significant’, **p*< 0.05, ****p*< 0.001.

**Supplementary Table S1. The sequences of the primers**

| **The shRNA template sequences** | | | |
| --- | --- | --- | --- |
| **Genes** | **Sequences (5' to 3')** | | |
| HPV18 E6-, #1 | AAAAGGTGCCAGAAACCGTTGAATCTGGATCCAAGATTCAACGGTTTCTGGCACC | | |
| HPV18 E6, #2 | AAAAACCTGATCTGTGCACGGAACTTTGGATCCAAAGTTCCGTGCACAGATCAGG | | |
| WNT4, #1 | AAAAGCGCTGTTCCTCCATGAAACTTTGGATCCAAAGTTTCATGGAGGAACAGCGC | | |
| WNT4, #2 | AAAAGGATCAAGACCTTGCCCTTGATTGGATCCAATCAAGGGCAAGGTCTTGATCC | | |
| JIP1, #1 | AAAAGGAGGAGTTTGAGGATGAAGATTGGATCCAATCTTCATCCTCAAACTCCTCC | | |
| JIP1, #2 | AAAAGCATCAGCTTACAGTGCAAAGTTGGATCCAACTTTGCACTGTAAGCTGATGC | | |
| JIP2, #1 | AAAAGGACATAAGCCTGGAAGAATTTTGGATCCAAAATTCTTCCAGGCTTATGTCC | | |
| JIP2, #2 | AAAAGCCATGCAGAAGATTGCCACTTTGGATCCAAAGTGGCAATCTTCTGCATGGC | | |
| **The primer sequences for cloning** | | | |
| **Genes** | **Forward primers (5' to 3')** | | **Reverse primers (5' to 3')** |
| HPV18E6 | CGACGCGTGCCACCATGGCGCGCTTTGAGGATC | | CTAGCTAGCTTATACTTGTGTTTCTCTGCGTC |
| WNT4 | GCTCTAGAGCCACCATGAGTCCCCGCTCGTGCCT | | CGACGCGTTCATCGGCACGTGTGCAACTC |
| JIP1 | GCTCTAGAGCCACCATGGCGGAGCGAGAAAGC | | CGGGATCCCTACTCCAGGTAGATATCTTCTGTG |
| JIP2 | GCTCTAGAGCCACCATGGCGGATCGCGCGGAG | | CGGGATCCCTACTCCAGGTAGATGTCCTCCGTG |
| **The primer sequences for qRT-PCR** | | | |
| **Genes** | | **Forward primers (5' to 3')** | **Reverse primers (5' to 3')** |
| HPV18E6 | | GCGACCCTACAAGCTACCTG | GTTGGAGTCGTTCCTGTCGT |
| WNT4 | | GAGCAACTGGCTGTACCTGG | ACACCTGCCGAAGAGATGGC |
| MAPK8IP1(JIP1) | | CCGCGGAGAGCGCCAT | GCTTTCTCGCTCCGCCATTC |
| MAPK8IP2(JIP2) | | GGGCGTGTCACGAGGTGAG | CCCAGGCCACAGTCATCAGT |
| STMN3 | | GGGACCACCCTTTGCGAC | AGCACCGACAGCTCCTTCATC |
| KCND3 | | AGCTGATTGTCCTCAACGTGA | TCGTCGTCGTAGGCAGAGAT |
| KCNQ2 | | TTGAGAAGAGGCGGAACCCG | ACGGGCTGCCTTTACTTGGA |
| CGA | | ACAGTCAACCGCCCTGAACA | ACCCGGCTGGGAGAAGAATG |
| ADAMTS8 | | AGAGGAGAAAGCGGTTGGGG | GCAAAACCAATCAGGCAGCG |
| ZCCHC24 | | ACCTTCTGGTTGAGCTGCAAGA | TCATCCAGGGAAAACAACACAGAA |
| CPLX1 | | CGGGAAGGAAGAACACTCGC | TACTTGTCTCGGATGCCCTGG |
| BICDL1 | | GCCTTCAGGCCGAGATCAAG | AGACGCACCTCCTGAAGCTC |
| CYFIP2 | | CACCCTGGAAGATGCCCTGT | CAGGTGTACAGCATGACCGC |
| CDK2AP1 | | CTTACAAACCGAACTTGGCCGC | CCCAGTTCCCTGGGTGTAGC |
| AKR1C1 | | AATTGAAGCTGGCTTCCGCC | ACCTTTCCAAGGCTGGTCGG |
| GDF15 | | GTTGCGGAAACGCTACGAGG | TCACGTCCCACGACCTTGAC |
| AKR1C2 | | CCATTGGAATGACATACTGCATCCT | TGAGAGGAGGGACAGAGGCA |
| SPINK6 | | ATGTGTTGGCAGGCCCCATTA | TGCAGTAGACCTTGGGGTCC |
| CEACAM7 | | CGTCACCCACAATGACGCAG | TGGCGCTGAGTAGAACGAGG |
| CA12 | | GTGCAAGTCTGTACTGCGGC | TCGCAAGTGTCCAGAGAGCC |
| SCARA5 | | CCATCGCACTGCGGAACATC | TTTGGGCCTCGGTCACCTTT |
| SEL1L3 | | TGCTCCTGCTCTGCTACCTG | TGTGTCCTGCTGGTGTGAAGA |
| CDKN1A | | CACCGAGGCACTCAGAGGAG | CCTCCTCCCAACTCATCCCG |
| HIST1H2BK | | ACAAGCGCTCGACCATCACC | GAGCCTTTGGGGTTGGGCTTT |
| ALOX5 | | GTGGCGCGGTGGATTCATAC | GCGTCCATCCCTCAGGACAA |
| NDRG1 | | CAGGACCGCCCTTTGTAGCC | AGCTGGGATTCGGAGACGAC |
| SPINK5 | | GAGTTCAGTCATACTGCACCAGC | TCTTATCCTGGGGACAGAACAGT |
| β-actin | | CGTCACCAACTGGGACGA | ATGGGGGAGGGCATACC |

**Supplementary Table S3. Silencing E6 caused fold changes on the translation efficiencies of know 5’ TOP and 5’ TOP-like mRNAs**

| **5’ TOP mRNAs** | |
| --- | --- |
| **Genes** | **Fold change of translation Efficiency: log2(shE6/SC)** |
| PABPC1 | -0.260751792 |
| EEF2 | 0.072634459 |
| EEF1A1 | -0.090657856 |
| RPS18 | 0.075630203 |
| EEF1B2 | 0.035357107 |
| TPT1 | -0.334848344 |
| RPS19 | 0.133847828 |
| RPS20 | 0.075274747 |
| RPS4X | 0.073123951 |
| RPL14 | 0.303732987 |
| RPL18A | 0.127966178 |
| RPL32 | 0.162138318 |
| RPLP0 | -0.089004619 |
| RPS24 | 0.265079153 |
| RPL13A | 0.06982148 |
| RPL34 | 0.188884679 |
| RPL4 | 0.13113596 |
| RPS15A | 0.079520004 |
| RPS5 | 0.090605353 |
| RPL10A | 0.275941693 |
| RPL22 | 0.180461717 |
| RPS26 | 0.232682974 |
| RPL35 | 0.435309281 |
| RPL7 | 0.372656132 |
| RPL8 | 0.374323161 |
| RPS9 | 0.383142479 |
| RPS10 | 0.172842824 |
| RPL12 | -0.170750141 |
| RPLP2 | 0.118827903 |
| RPS3A | 0.137916713 |
| EIF3F | 0.033923398 |
| EEF1G | 0.181894372 |
| RPL39 | -0.151768554 |
| RPS3 | 0.199545664 |
| EIF3H | 0.357462155 |
| RPL6 | 0.238588739 |
| RPL9 | 0.256173733 |
| RPL23 | 0.286268073 |
| RPL7A | 0.156757782 |
| RPS25 | 0.045543709 |
| RPS14 | 0.158655971 |
| RPS16 | 0.084458247 |
| RPL24 | 0.313140821 |
| RPL37 | 0.233456822 |
| RPS15 | 0.310375374 |
| RPS21 | 0.074850652 |
| RPL18 | 0.330165242 |
| RPL21 | 0.025309861 |
| RPS11 | 0.009321543 |
| FAU | 0.283989853 |
| RPL5 | 0.199878229 |
| RPLP1 | -0.108076145 |
| RPL23A | 0.025802769 |
| RPL37A | 0.083008816 |
| NPM1 | 0.709940781 |
| RPL38 | 0.206431902 |
| RPL30 | 0.195365065 |
| RPL41 | 0.063993836 |
| RPL19 | 0.220339226 |
| RPL36A | 0.310864784 |
| EIF3E | 0.342371801 |
| EEF1D | 0.401427488 |
| EEF1E1 | 0.377891033 |
| HNRNPA1 | 0.354599716 |
| RPS27A | 0.20193414 |
| **5’ TOP-like mRNAs** | |
| YBX1 | 0.112414 |
| ODC1 | 0.574171 |
| WDR89 | 0.177483 |
| NME2 | 0.712824 |
| VIM | 0.281443 |
| COL4A1 | -0.40865 |
| GYG | 0.531786 |
| GLUD1 | -0.21211 |
| TAF10 | 0.130113 |
| NUP35 | 0.551115 |
| SRP72 | -0.14629 |
